# Impact of the COVID-19 Pandemic on Patient- and Family-Centered Care and on the Mental Health of Health Care Workers, Patients, and Families

**DOI:** 10.3389/fped.2022.880686

**Published:** 2022-07-12

**Authors:** Alessandra Rodrigues Dias Lessa, Victória Noremberg Bitercourt, Francielly Crestani, Gabriela Rupp Hanzen Andrade, Caroline Abud Drumond Costa, Pedro Celiny Ramos Garcia

**Affiliations:** Graduate Program in Pediatrics and Child Health, School of Medicine, Pontifícia Universidade Católica do Rio Grande do Sul (PUCRS), Porto Alegre, Brazil

**Keywords:** intensive care units, pediatrics, children, health care, humanization of care, family-centered care

## Abstract

During the COVID-19 pandemic, hospitals around the world were forced to reorganize their processes in an attempt to contain the spread of the virus while still providing adequate care to patients. In the Pediatric Intensive Care Unit (PICU) setting, changes in family visitation protocols and restrictions on parent chaperones during hospitalization, as well as other changes, interfered with care. Based on a narrative review of the literature, supported by the authors' observations in practice, we aimed to describe the impact of the pandemic on patient and family-centered care (PFCC) in the PICU environment, especially regarding the presence of family members, family support, and communication with patients and their families, as well as the effects of changes in these practices on the mental health of those involved. In this context, several strategies were used to sustain PFCC, and, despite many challenges, attempts were made to achieve the bare-minimum goals of humanized care for patients, families, and providers alike.

## Introduction

The novel coronavirus (SARS-CoV-2) spread rapidly worldwide, resulting in a pandemic being declared in March 2020 (1). Children represent only 1 to 5% of all cases of coronavirus disease 2019 (COVID-19) overall (2, 3) and generally experience a milder clinical condition compared to adults, with few reported deaths (4). One study found that, among pediatric cases, 14.2% of diagnosed patients were asymptomatic, 36.3% had mild symptoms, 46% moderate, 2.1% severe, and 1.2% were critically ill, with only one death reported (5). Infants and young children experience more severe disease than older children do (6). Of the patients who required intensive care, some developed severe acute symptoms, especially those with comorbidities; however, most had good outcomes with the treatment provided (7, 8).

As the disease spread worldwide and aiming to ensure the continuity of adequate care, the need arose to restructure and reorganize patient management protocols. As health care workers, families, and patients were forced to practice physical distancing, the relationship between them and the practices involved in patient- and family-centered care (PFCC) were affected directly and indirectly (9). Based on a narrative review of the literature, and supported by our observations in clinical practice, we aimed to describe the impact of the pandemic on PFCC in the PICU environment, especially regarding the presence of family members, family support, and communication with patients and their families, as well as their effects on the mental health of those involved.

### PFCC: An Overview

PICU care has evolved substantially in recent years, both in terms of technology and humanization of care. Visits and the presence of parents at the bedside during a child's hospital stay were long forbidden. After World War II, with the mass separation of children from their parents, several studies emerged on the psychological effects of this separation during childhood, including hospital admissions (10). However, until the mid-20th century, children were still separated from their parents during hospitalization. At this time, a growing movement sought to raise awareness of the importance of meeting the psychosocial and developmental needs of children, as well as of the importance of the family in promoting child health and well-being. As research was published demonstrating the effects of separation of children from their families, many facilities began to adopted policies that encouraged parents to remain at their children's side during hospitalization and medical procedures (11).

The perspectives and information provided by the child's family play an important role in clinical decision-making, considering that the family is the main source of strength for the child (12). In addition, some pediatric patients are not able to verbalize their symptoms and desires, either due to their stage of development or their clinical condition, leaving parents in charge of communicating on behalf of the child and thus making collaboration between parents and health care workers particularly important (11).

In an attempt to change the way providers care for and interact with children and families, PFCC—a health care practice based on respect for individual needs and values (13), which encompasses comprehensive care and guides patient and family autonomy in care and decision-making (12)—was introduced in pediatrics. PFCC is an approach to planning, implementing, and evaluating health care based on collaboration between providers, patients, and the family, for the benefit of all; its core tenets are dignity and respect, information sharing, participation, and collaboration (14). These approaches lead to better health outcomes and wiser resource allocation, as well as greater stakeholder satisfaction (12). In Table 1, we cite some of the studies that address patient- and family-centered care.

**Table 1:** Articles on PFCC in PICU.

**Title**	**Authors**	**Year**	**Local**	**Design**	**Main findings**
Exploring the experiences of parent caregivers of children with chronic medical complexity during pediatric intensive care unit hospitalization: an interpretive descriptive study (15)	Janet E Rennick et al. (3)	2019	Canadá	Interpretive descriptive study	Need for a different approach to PICU care for chronic medical complexity, with an emphasis on establishing parent-staff partnerships to optimize patient care; Parents were vigilant about their child's comfort, noting the importance of reminding staff of the child's unique sensitivities and needs; While they felt they played an important role, parents did not always feel welcome; Parents struggled when physicians made decisions without consulting them, when information they provided about their child's preferences and needs was not acknowledged, or when the team did not apprise them of changes in the child's care plan; The needs expressed by parents of chronic medical complexity during PICU hospitalization included enhanced partnerships with health care professionals, improved communication with staff, and more attention to continuity of care in the PICU and across hospital services.
Nurses' reflections on benefits and challenges of implementing Family-centered care in pediatric intensive care units (16)	Heather Coats et al. (7)	2018	EUA	Qualitative description	Family-centered care brings benefits to parents, but it also creates many challenges for the team; The two main changes to this care are ICU policies related to visiting hours and family presence at the bedside and (2) transformation of the ICU's physical environment from a shared open space to individual private rooms; Nurses play a key role in the implementation of PFCC.
Elements of family-centered care in the pediatric intensive care unit: an integrative review (17)	Claire A. Richards et al. (3)	2017	EUA	Integrative review	Were identified 5 main themes related to Family Centered Care: 1) sharing information with parents. 2) hearing parental voices. 3) making decisions for or with parents. 4) individualizing communication; and 5) negotiating roles; There are gaps between parents' expectations of their involvement and how much they perceive that they are involved in the care of their child; Clinicians still own information and determine how much information parents will have access to, how much they will participate in decisions, and when they will be involved in procedures; Asking parents about their expectations regarding communication and their participation can improve doctor-family relationships, patient care, reduce conflicts and alleviate emotional distress.
A narrative synthesis of the components of and evidence for patient- and family-centered care (18)	Kaitlin P. Gallo et al. (3)	2017	EUA	Narrative synthesis	The PFCC has a positive impact on patient and/or family behavior, experience, knowledge and attitudes of patients/family, provider behavior and health status; The relationship of the individual components of the PFCC and the results showed that socio-emotional support to the patient or family was associated with positive changes in the patient/knowledge, attitudes and/or family experience.
Parent satisfaction with communication is associated with physician's patient-centered communication patterns during family conferences (19)	Tessie W. October et al. (5)	2016	EUA	Cross-Sectional study	Patient-centered communication scores higher when topics are related to psychosocial, lifestyle, and socio-emotional focus vs. medically focused conversation; Parental satisfaction is significantly higher the more the communication is patient-centered; The severity of the patient's illness were the factors influencing and maintaining parental management.
Models of care delivery for families of critically Ill children: an integrative review of international literature (20)	Kate Curtis et al. (3)	2015	Australia	Integrative review	None of the care models analyzed offers intervention throughout each phase of care until (or after) hospital discharge, but at a specific stage of it; The models of care evaluated all had a positive impact on enhancing families' and parents' experience in a paediatric setting; Models of care applying only one or two aspects of approaches such as FCC, shared care, partnered care and increased caregiver involvement in care provision of critically ill children were associated with reduced parental anxiety, increased parental satisfaction in care provided and improved communication between parents and health care providers.
Family-Centered care in the pediatric intensive care unit (21)	Kathleen L. Meert et al. (3)	2013	EUA	Narrative review	Preliminary research on the implementation of programs related to PFCC in the PICU setting generally suggests benefits to patients, families, and staff; The development of PFCC policies and their implementation in clinical practice should reflect the needs of specific patient populations and settings, and thus requires continued input from patients, families, and staff; Patient-centered communication is the ideal process through which PFCC is implemented in daily practice.

PFCC guidelines include having the family present in the PICU, providing family support, and communicating with family members (13). The pandemic and the attendant increase in COVID-19 cases have led to economic, social, and political impacts in several countries, as well as implications for the availability of hospital beds, equipment, and other health resources (22). In order to contain the spread of the pandemic, several restrictive and restructuring measures were implemented, including in hospitals, with impacts on PFCC and on the mental health of those involved.

### Family Presence in the PICU

The U.S. Centers for Disease Control and Prevention (CDC) advises that visits to health facilities be limited during the pandemic. Visits to patients who are in COVID-19 isolation should be restricted to those who provide essential care, such as parents, with only one person allowed at a time. The presence of family members during aerosol-generating procedures or respiratory specimen collections is contraindicated (23). In most PICU settings, all visits were banned, only one parent or legal guardian was allowed to remain with the child and, even so, for shorter periods of time (24). At some hospitals, older children and adolescents were not allowed a chaperone when they were sedated. In addition to these restrictions, other issues can prevent parents from being with their children, such as COVID-19 infection itself, which requires isolation of the infected parent and reorganization of the family unit so that another responsible adult can chaperone the child.

Maintaining social distancing from one's family during hospitalization can lead to greater parental vulnerability, threaten parental autonomy, and risk returning to a disease-centered approach, neglecting patient- and family-centered care (25). Studies carried out before the pandemic showed that more than half of all parents of children admitted to a PICU experienced symptoms of anxiety or depression during their child's hospitalization, which remained for approximately 3 months after discharge (26). Of these, 10.5 to 21% were diagnosed with post-traumatic stress disorder (PTSD) and 84% continued to experience symptoms of this disorder after their child's discharge from the PICU, with mothers being most affected. Among children, 5 to 28% had a diagnosis of PTSD after PICU admission, and 35 to 62% had symptoms of PTSD (27).

Another study showed that parents of children hospitalized during the pandemic had significantly higher rates of anxiety and depression symptoms than parents of children hospitalized before the pandemic period (28). Children who have lost a parent to COVID-19 are more susceptible to mental health problems due to fear of circumstances and grief over the loss of the parent (29).

For children in palliative care and their families, visits are particularly important. Restricting visits in these cases increases anxiety, suffering, and moral damages, and negatively impacts patient quality of life (30, 31). In addition, the suffering of family members of children who die in the PICU can be made worse by being away from their support network (31).

The presence of family members during PICU hospitalization is essential to minimize the emergence of psychological symptoms in children and parents alike, both during hospitalization and after discharge. Given the current situation, new ways of providing PFCC are needed. Virtual meetings have been the strategy used in some facilities to bring parents closer to their children, sharing decision-making for care and minimizing the negative impacts of separation (9).

### Family Support

Family support refers to the support provided by health care workers to family members on issues including family education, involvement in care, use of tools for assertive communication and support of family decisions, as well as respect, recognition, and acceptance of the family's values and emotions when faced with a poor prognosis or the death of the patient, aiming to improve the mental health of parents during and after PICU discharge and increase family satisfaction with care (13).

Studies have shown that parents are satisfied with the care provided in PICU settings, especially with regard to providers' attitudes (32–34). During the COVID-19 pandemic, we have found that many health care workers have also been affected psychologically due to increased workload, fear of contamination, and shortage of human and material resources, among other factors (35). The risk of contagion, absence from work when infected, and even witnessing the deaths of colleagues and patients have created additional stress for many health care providers (36). The idiosyncrasies of health care work compound the aforementioned issues and include being undervalued, poor working conditions, unsafe physical infrastructure, inadequate staffing, poor flow along care pathways, lack of cooperation for teamwork, and support from leaders, among others (35).

The prevalence of mental disorders in front-line providers is expected to rise, as psychological disorders such as anxiety, depression, and PTSD have already been identified in health care workers after previous pandemics (37). Among nurses who worked directly in the care of patients with severe acute respiratory syndrome (SARS) during the 2003 outbreak, the development of psychiatric symptoms was linked to direct exposure to SARS patients, history of previous mood disorder, younger age, and perceived negative feelings (38).

In addition to the difficulty of providing support due to the absence of other family members at the PICU, we believe that the mental health of providers themselves also interferes with this practice, considering that they are also emotionally distressed and experiencing the consequences of the pandemic on their personal lives.

Availability of counseling within the hospital is of paramount importance to support the fight against COVID-19, as well as to support children and parents during treatment and hospitalization. The pandemic has hindered the provision of psychological and spiritual support to families (31). However, virtual care has been a method of providing support and helping to cope with the situation, with many services offered free of charge to the population (29, 39).

Several health facilities have also invested in hiring mental health teams to support their personnel, working to prevent and treat symptoms arising from the burden of working in a pandemic context.

### Communication With Patients and Families

Communication between health care workers, family members, and patients is a core feature of PFCC. Interdisciplinary meetings with the family help ensure assertive communication, increase trust in the team, and improve family satisfaction. Active listening and expressions of empathy and support must all be present in communications (13). The use of personal protective equipment (PPE) such as masks and gowns has been recommended both inside and outside the hospital. However, these interfere with nonverbal communication, which is of the utmost importance in interactions between providers, patients, and families, and can make it difficult for health care workers to gauge the emotional reactions of family members, of which they must also be aware (9, 40).

Shared decision-making between physician, patient, and family is at the heart of communication in PFCC. However, during the pandemic, difficult situations—such as the need to decide which patients will receive treatment and which will be denied due to limited resources—cause ethical stalemates, with psychological repercussions for health care workers; this interferes with provider-patient-family communication (22, 41).

The high workload of health personnel and the absence of family members at some moments during hospitalization have also interfered with communication. In this context, telemedicine has been used as a strategy to maintain communication with families, such as by holding video calls with parents during rounds and scheduling meetings to share decisions and care plans (9). Although not equivalent to face-to-face encounters (42), these strategies can minimize difficulties in communication.

## Discussion

The COVID-19 pandemic has imposed several organizational changes in hospital settings, as well as the implementation of new contingency protocols to ensure continuity of patient care while containing the spread of the virus. The need to restrict visitation of PICU patients during the COVID-19 pandemic is understandable, considering the high transmissibility of the virus, the scarcity of PPE, and the lack of knowledge about the course of the disease (42). However, there were difficulties in finding a balance between containing the spread of the disease and maintaining the humanization of care, through such strategies as allowing the presence of family members (31).

PFCC is the gold standard of care in pediatrics. Considering the importance of family presence at the bedside in PICU settings while respecting guidelines to control the spread of the virus, most institutions generally found ways to allow at least one family member to remain with the child during hospitalization, which helps both child and parent face the situation and, in the event of the patient's death, allows for a better working through of bereavement (42).

The repercussions of the pandemic directly and indirectly affect patients, families, and health personnel; all have suffered physical and emotional consequences. It is essential that support be available to all, at all levels, in order to prevent and address harmful impacts (41). Comprehensive care in the PICU environment requires a holistic bio-psycho-social understanding of the subject; the pandemic has created stress at all of these levels. Thus, patients hospitalized during this period and their family members were exposed not only to the habitual stress of being in hospital or having a child in hospital but also to various other concerns inherent to the pandemic period, such as unemployment, financial difficulties, bereavement, and social isolation, among others, which reflect on experiences during hospitalization.

One of the main ways to maintain contact between family and health care workers, as well as between patients and their relatives, has been the use of technology. Video calls for virtual visits and meetings with multidisciplinary teams were implemented all over the world. However, some families had limited access to a reliable internet connection and electronic devices that support video calls, among other difficulties (43).

Medical news was often communicated by phone or video calls, with a negative impact on family support, as this hindered perceptions of and responses to the families' emotions.

Having the family present is a core element of PFCC. We found evidence that several strategies were used to bring families closer and ensure their participation in care, and believe that also had positive impacts on medical teams, with families as allies in patient care (42).

Drastic changes in care processes were required during this period, and substantial efforts were needed to maintain PFCC. The long-term consequences of the pandemic on the lives of patients, families, and health care workers remain unclear.

## Author Contributions

AL and PG contributed to the conception or design of the study and acquisition, analysis or interpretation of data, wrote the manuscript, critically reviewed the manuscript, gave final approval, and agrees to be responsible for all aspects of the work ensuring integrity and accuracy. VB, GA, FC, and CC wrote the manuscript, critically reviewed the manuscript, gave final approval, and agrees to be responsible for all aspects of the work ensuring integrity and accuracy. All authors contributed to the article and approved the submitted version.

## Funding

The present study was supported by the Brazilian funding agency CAPES (Coordenação de Aperfeiçoamento de Pessoal de Nível Superior).

## Conflict of Interest

The authors declare that the research was conducted in the absence of any commercial or financial relationships that could be construed as a potential conflict of interest.

## Publisher's Note

All claims expressed in this article are solely those of the authors and do not necessarily represent those of their affiliated organizations, or those of the publisher, the editors and the reviewers. Any product that may be evaluated in this article, or claim that may be made by its manufacturer, is not guaranteed or endorsed by the publisher.

## Nomenclature

### Resource Identification Initiative

To take part in the Resource Identification Initiative, please use the corresponding catalog number and RRID in your current manuscript. For more information about the project and for steps on how to search for an RRID, please click here.

### Life Science Identifiers

Life Science Identifiers (LSIDs) for ZOOBANK registered names or nomenclatural acts should be listed in the manuscript before the keywords with the following format:

urn:lsid: <Authority>:<Namespace>:<ObjectID>[:<Version>] For more information on LSIDs please see Inclusion of Zoological Nomenclature section of the guidelines.
